# The Rise and Fall of Dopamine: A Two-Stage Model of the Development and Entrenchment of Anorexia Nervosa

**DOI:** 10.3389/fpsyt.2021.799548

**Published:** 2022-01-11

**Authors:** Jeff A. Beeler, Nesha S. Burghardt

**Affiliations:** ^1^Department of Psychology, Queens College, City University of New York, Flushing, NY, United States; ^2^Psychology Program, The Graduate Center, CUNY, New York, NY, United States; ^3^Biology Program, The Graduate Center, City University of New York, New York, NY, United States; ^4^Department of Psychology, Hunter College, CUNY, New York, NY, United States

**Keywords:** anorexia nervosa, compulsive behavioral disorders, dopamine, chronic stress, behavioral plasticity

## Abstract

Dopamine has long been implicated as a critical neural substrate mediating anorexia nervosa (AN). Despite nearly 50 years of research, the putative direction of change in dopamine function remains unclear and no consensus on the mechanistic role of dopamine in AN has been achieved. We hypothesize two stages in AN– corresponding to initial development and entrenchment– characterized by opposite changes in dopamine. First, caloric restriction, particularly when combined with exercise, triggers an escalating spiral of increasing dopamine that facilitates the behavioral plasticity necessary to establish and reinforce weight-loss behaviors. Second, chronic self-starvation reverses this escalation to reduce or impair dopamine which, in turn, confers behavioral inflexibility and entrenchment of now established AN behaviors. This pattern of enhanced, followed by impaired dopamine might be a common path to many behavioral disorders characterized by reinforcement learning and subsequent behavioral inflexibility. If correct, our hypothesis has significant clinical and research implications for AN and other disorders, such as addiction and obesity.

Worldwide, millions of people diet and struggle to lose weight, with high rates of relapse and weight rebound. This is not surprising considering that we evolved over millions of years to eat, a behavior that is highly reinforcing. Yet, evolution and a lifetime of reinforcement are seemingly overridden in a subset of dieters who develop anorexia nervosa (AN), an eating disorder characterized by unrelenting self-starvation.

Initially, AN behaviors may be indistinguishable from a successful weight loss routine. AN becomes evident when an inability or unwillingness to *stop* losing weight emerges, even when it is life threatening. Those with AN develop an obsessive preoccupation with being thin, often engaging in ritualistic, restrictive eating behaviors and vigorous, extended exercise. The nature of these behavioral changes have been compared to compulsions in obesity and addictive disorders [e.g., (1)], where entire patterns of behavior become reorganized around a central focus (i.e., weight-loss, overeating, drug taking) often interfering with other activities and social relationships. As in obesity and addiction, once a behavioral regimen has been established in AN, it can be highly resistant to change, or entrenched (2).

The neuroadaptations underlying AN remain poorly understood. Starvation alone leads to physiological and psychological changes that resemble AN symptoms (3–5) and unintended weight loss has been found to trigger AN in some cases (6). This has led to the suggestion that dieting and exercising – typically motivated by psychosocial and cognitive factors – could lead to weight loss-induced adaptations that trigger AN (7, 8). However, this fails to account for the most crucial aspect of AN, the *refusal* to eat. After all, a starving person will generally eat when offered food. It is important to understand 1) how weight loss leads to the reorganization of behavior around *self-starvation* in some individuals and 2) the mechanisms by which these behaviors become compulsive and entrenched.

The midbrain dopamine system has been implicated in the pathophysiology of AN for decades (7, 9, 10). However, consensus on *how* dopamine mediates AN has yet to emerge. We propose a two-stage model of AN in which opposite changes in dopamine function underlie each stage of the disorder: initial emergence and establishment of AN behaviors, and subsequent entrenchment of an established AN behavioral regimen.

## Dopamine in Anorexia Nervosa

The hypothesis that increased dopamine plays a central role in the pathophysiology of AN was first introduced in 1976 by Barry and Klawans (11). This was based on the observation that drugs that increase dopamine, such as amphetamine, lead to changes that resemble AN symptoms. Subsequent studies measuring dopamine or its metabolites reported increased, decreased or unchanged levels in AN (9, 12). Human imaging studies using PET revealed increased D2/3 binding in the ventral striatum of recovered AN patients (13), which could reflect increased receptor expression, decreased dopamine transmission, or both if D2/3 is upregulated in response to diminished basal dopamine. In contrast, Broft et al. (14) found no change in D2 availability in currently ill AN patients. In a subsequent fMRI study using a prediction error task, Frank et al. (15) observed enhanced activity in striatal and insula regions, consistent with enhanced dopaminergic responsivity in AN. Collectively, this work supports a role for abnormal dopamine signaling in AN, but the *direction* of the abnormality remains unclear.

In the absence of longitudinal, prospective studies, it is difficult to determine whether putative abnormalities in dopamine precede the disorder representing a risk factor or arise as a consequence of starvation. In a widely cited paper, Kaye et al. (16) reported that recovered women with restricting-type AN have reduced homovanillic acid, a major metabolite of dopamine. While one interpretation of this result is that there is trait-like dysfunction in dopamine metabolism, it is also possible that this reflects persistent changes induced by AN. Recovered patients often continue to exhibit AN characteristics even though they no longer fulfill diagnostic criteria (13, 17–20). Therefore, changes in dopamine originally induced by AN could persist after recovery and underlie residual symptomatology. Genetic studies have also not clarified whether pre-existing variation in dopamine constitutes a risk factor for AN. While there have been reports of associations between dopamine related genes and AN, none of these have been consistently replicated and none have been confirmed in large, genome-wide association studies (21–23). Thus, whether altered dopamine represents a risk factor preceding AN or a pathophysiological adaptation arising as a consequence of AN remains unclear.

The results of animal studies have been more consistent with Barry and Klawan's hypothesis that overactive dopamine drives AN. Caloric restriction in rodents has been associated with increased dopamine sensitivity and function (24–26). In activity-based anorexia (ABA), a rodent model of AN that combines food restriction with wheel running (27), antipsychotics that decrease dopamine signaling by blocking D2R have been shown to limit weight loss (28, 29). Unfortunately, the antipsychotic drugs used have effects on activity, motor ability, motivation, and metabolism, confounding interpretation of the results. Furthermore, antipsychotics have not been efficacious in treating AN in humans (30), although there is some evidence that atypical antipsychotics, such as olanzapine, may be effective as a treatment augmentation strategy (31–34). However, a review of randomized controlled trials found insufficient evidence to support atypical antipsychotics as a standard treatment for AN (35).

A role for enhanced dopamine signaling in AN is partially supported by other ABA studies using more targeted approaches. For example, mice that increase dark cycle running across days of food restriction demonstrate an upregulation of D2R expression in the striatum (36), consistent with increased D2/3 binding observed in recovered patients (13). Selective pharmacological blockade of D2R reduces vulnerability to ABA (28), while genetic overexpression of D2R in the nucleus accumbens core increases ABA vulnerability (37). Similarly, we reported that hyperdopaminergia resulting from knockdown of the dopamine transporter in mice also enhances ABA vulnerability (38). Furthermore, the only study to directly measure dopamine during ABA with microdialysis (29) found increased dopamine release in the nucleus accumbens during food intake in rats. However, no changes in dopamine were detected prior to food availability, which is when wheel running progressively increases in some ABA animals (i.e., food anticipatory activity), and dopamine was actually *decreased* during the light cycle (29). Foldi et al. (39) used chemogenetics to directly target dopamine cells in the mesolimbic pathway during ABA and found that activation of G_q_ coupled DREADDs in the ventral tegmental area with systemic administration of clozapine-N-oxide (CNO) rescued the ABA phenotype, suggesting that impaired dopamine signaling is a driver of ABA. However, DREADD activation did not exclusively affect dopamine cells and protection against ABA could be attributed to activation of GABAergic projections to the nucleus accumbens. In addition, it is now known that systemic CNO is converted to the antipsychotic clozapine (40), which affects appetite and weight gain via mechanisms that may be independent of the targeted pathway. Notably, the Foldi et al. (11) finding suggests that drugs that increase dopamine, such as amphetamine, could treat AN, which is the opposite of the original Barry and Klawans hypothesis.

In sum, accumulated evidence in both humans and animal models indicates that dopamine is altered in AN, but characterizing this abnormality and its contribution to AN symptomology remains an unresolved challenge.

## Hypothesis

Dopamine has been studied extensively in addiction research, where there is also a question of whether the core problem is increased or decreased dopamine. In that debate, a critical distinction can be made between the acute effects of drugs, which are known to cause increased dopamine release, and the more complex, progressive changes in dopamine that occur over time as the brain adapts to chronic drug use. The nature of these progressive changes is controversial, with evidence for both impaired, diminished dopamine function and sensitized dopamine responses to drugs and drug-related stimuli (41).

Here, we incorporate this idea that dopamine changes progressively over time into our hypothesis of AN. We propose a pattern of first enhanced and then diminished dopamine function, corresponding to a gain and then a loss of behavioral flexibility. This results in two stages in the development of AN, each mediated by different underlying neural mechanisms.

### Stage 1: Initial Development of Anorexia Nervosa

**Figure 1:**
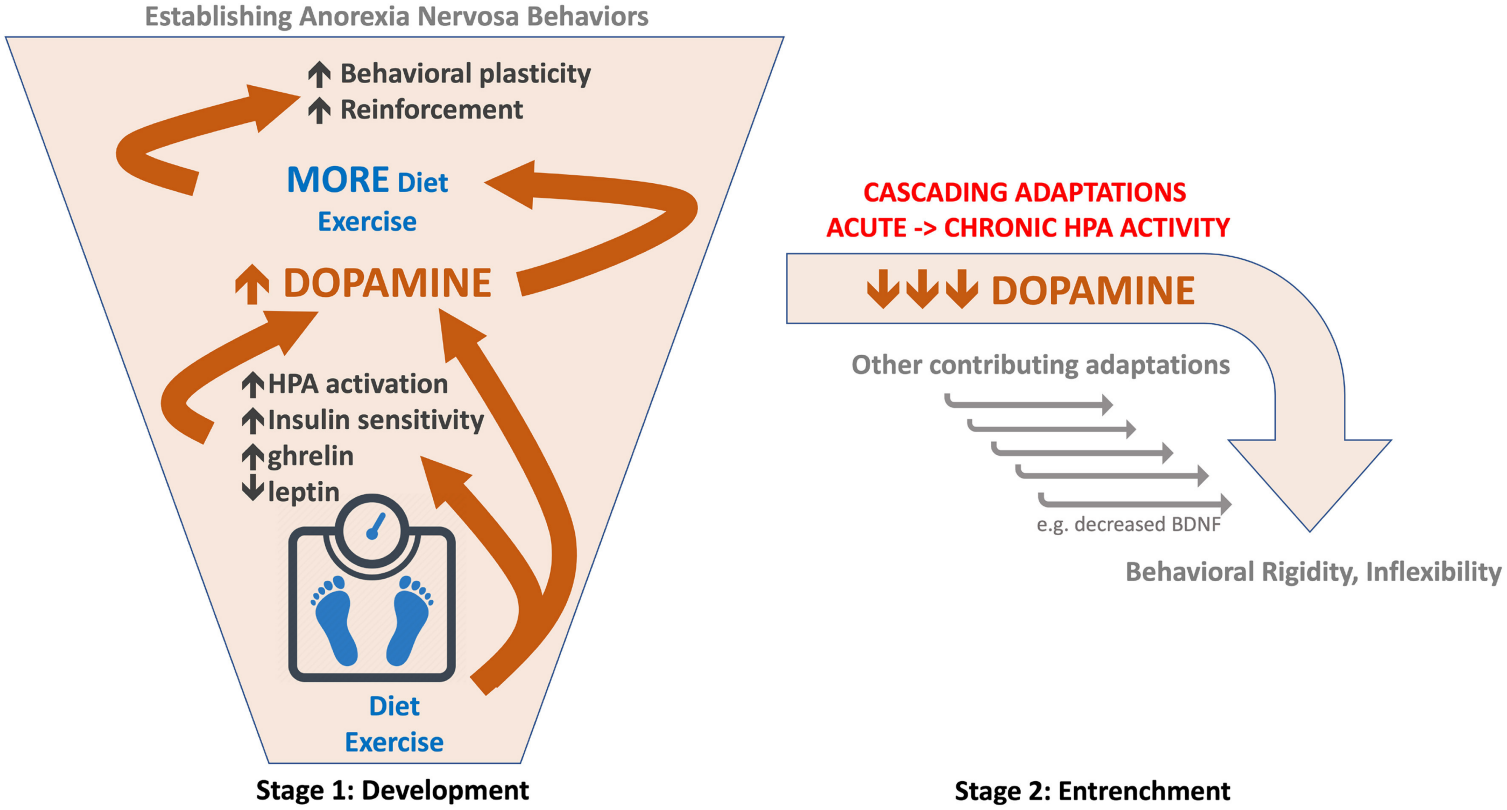
Schematic of hypothesized role of dopamine in two-stage model of anorexia nervosa. (Left) Stage 1: Development of AN. Diet and exercise trigger an escalating spiral of increased dopamine function (orange arrows and bounding box). This facilitates behavioral plasticity and reinforcement required for establishing a consistent self-starvation weight loss behavioral regimen. (Right) Stage 2: Entrenchment of AN behaviors. Persistent caloric deficit leads to a reversal in dopamine (orange) from augmented to reduced or impaired function; hypothesized as part of a cascade of adaptations resulting from chronic HPA activation.

We propose that in stage 1 of the disorder, weight loss resulting from caloric restriction triggers an increase in midbrain dopamine signaling, particularly when combined with high levels of physical activity. This increase could be mediated by stress-induced activation of the HPA axis (42), increased insulin sensitivity (43), decreased leptin (44), altered ghrelin (45), and/or other mechanisms (Table 1). As originally suggested by Barry and Klawans (11), the resulting increase in dopamine acts like a psychostimulant fueling both caloric restriction and exercise, which further augments dopamine signaling in an escalating spiral, creating a ‘dopamine storm' (Figure 1A). This escalation in dopamine facilitates reinforcement learning and behavioral plasticity necessary for establishing AN behaviors, as originally suggested by Södersten et al. (100). As a result, eating and activity routines are reorganized around achieving persistent weight loss. Individuals in stage 1 might be difficult to distinguish from non-anorexic dieters. As problematic behavior emerges, some individuals may receive EDNOS diagnoses during this earlier stage of development.

**Table 1:** Potential modulators of dopamine in each stage of AN.

**Modulators of DA**	**AN stage 1: enhanced DA**	**AN stage 2: diminished DA**	**Notes**
Caloric restriction	↑ DA burst activity, ↑ glutamate transmission onto midbrain DA cells (24) ↑ insulin enhancment of DA release (46)	↓ TH, ↓ EPSCs in VTA DA cells, ↓ evoked DA release (47) ↓ extracellular DA in NAc (48) ↓ glutamate transmission in VTA DA cells (49) ↓ evoked DA (46) ↓ basal DA, ↑ receptor sensitivity to phasic burst activity (26, 50)	Most preclinical work involves chronic and substantial food restriction and is thus most relevant for stage 2. Branch et al. (24) looked at mild food restriction, which is more comparable to early weight loss in stage 1 of AN. Collectively, the findings indicate that effects are dependent on the degree of weight loss, consistent with progressive changes in DA underlying stage 1 and 2.
Exercise	↑ striatal D2 (51, 52) ↑ TH mRNA, ↓ D2 autoreceptor, ↑ postsynaptic D2 (53) ↑ striatal D2 in abstinent methamphetamine users (54) ↑ cocaine (DA) reinforcement (55) ↑ DA response to insulin (43) ↑ DA response to stress (56) *Comment*: ↑ of striatal D2 would increase activity (57, 58) and facilitate synaptic plasticity (59–61)	*Comment*: Duration of exercise is variable across studies and the distinction between “acute” and “chronic” exercise is ill defined. However, because exercise affects several modulators of dopamine, such as the HPA axis, metabolism, weight loss and insulin sensitivity, exercise during persistent, sustained caloric restriction likely contributes to mechanistic changes underlying stage 2.	
Stress-acute	↑ DA via reuptake (62) > ↑ extracellular DA (56, 63) ↑ glutamate transmission at midbrain DA cells (64, 65) ↑ DA cell firing (66) ↑ glutamate induced burst firing (67)	Not Applicable	
Stress-chronic	Not Applicable	↓ tonic DA but ↑ DA cell responsiveness to glutamate transmission (42, 68) ↓ DA response to cocaine (69) ↓ DA cells (70) ↑ D2 in NAc (71) Note: as above, reflecting compensatory upregulation)	The effects of acute and chronic stress are widely known to be different. Less is known about the effects of chronic stress on dopamine, but the weight of evidence points to ↓ DA, likely with compensatory ↑ in receptor sensitization facilitating response to phasic/burst activity. Such changes are commonly thought to promote previously learned behavior.
Insulin	hypoinsulimea ↓ brain reward threshold (reflecting ↑ reward function) (72) astrocytic IR ↑ DA *via* purinergic signaling, likely ↑ probability of DA release (73) insulin ↑ cell autonomous (intrinsic properties) firing rate of DA cells, ↑ TH and D2 autoreceptors (74) insulin ↑ evoked DA (correlates with insulin sensitivity) (46) food restriction -> ↓ insulin -> ↑ insulin sensitivity -> ↑ DA release (75) ↑ D2 (from above factors) may ↑ insulin sensitivity (76)		
Leptin	acute leptin ↓ DA firing and ↓ glu transmission to VTA (77, 78) (*inference*: ↓ leptin associated with weight loss -> ↑ DA firing and glutamatergic drive) ↓ rewarding effect of running (44) (*inference*: ↓ leptin could facilitate reward associated with physical activity, contributing to caloric restriction -> activity escalation; Figure 1A)	chronic leptin deficiency ↓ TH, ↓ evoked DA, ↓ presynaptic DA stores (79)	
Ghrelin	↑ phasic DA (45) ↑ extracellular DA (80) acute fasting ↑ ghrelin sensitization (81)	Chronic elevated ghrelin ↓ ghrelin sensitivity (ghrelin resistance) (82) Opposite acute vs. chronic ghrelin-associated stress responses (83)	
Orexin (OR)	↑ extracellular DA (84) (*inference*: caloric restriction -> ↑ OR -> ↑ DA) blocking OR1 ↓ tonic DA, applying OR ↑ glutamate transmission to VTA, ↑ tonic DA, ↑ DA cell response to glutamate (85) ↑ LTP of excitatory synapses onto DA cells (86) ↑ DA firing, ↑ synaptic efficacy and ↑ DA neuron output (87) ↑ physical activity, potentially fueling spiral in Figure 1A (88, 89)	OR and HPA activation (CRF) interact in the VTA (90) (*inference*: Effects of OR may be altered as HPA activation becomes chronic).	OR ↑ under glucoprivic conditions (91–95)
Estrogen/estradiol	↑ phasic DA in dorsolateral striatum (96) ↑ DA response to amphetamine and ethanol (97, 98) (*Inference*: Estrogen may augment dopamine responsiveness)	hormone replacement ↑ reward activity in menopausal women (99) *Comment*: Enhancing effects of estrogen on DA may diminish/abate with prolonged amenorrhea and associated estrogen deficiency, possibly contributing to DA deficiency	Many of the observed effects are on dorsolateral striatum, believed to be a key substrate for habitual behavior

Increased physical activity was recognized as a characteristic of AN in its earliest description (101) and is observed in up to 80% of patients (102, 103). Adolescent girls who develop AN tend to exhibit higher premorbid activity levels (104) and athletes are at higher risk for developing AN (105). Vigorous exercise may contribute to the development of AN by driving dopamine escalation, thereby accelerating development of the disorder (38).

Midbrain dopamine projects to and receives afferents from several brain regions implicated in AN, including the prefrontal cortex, insula, hippocampus, amygdala, and hypothalamus (106). Weight loss-induced changes in these regions could contribute to the proposed escalation in dopamine through their afferent projections to the midbrain. Conversely, as targets of dopamine, escalating dopamine activity could affect plasticity and processing in these same regions. Dopamine is thus situated to be an engine driving a cascade of neuroadaptations across the brain [e.g., (107)].

### Stage 2: Entrenchment of Anorexia Nervosa

Stimuli effective at releasing dopamine, such as drugs of abuse and palatable food, can paradoxically reduce dopamine function with chronic, repeated exposure (108, 109). We propose a similar pattern in AN where the escalating dopamine spiral 'collapses' following long-term caloric restriction and dopamine becomes impaired (Table 1). In contrast to the behavioral flexibility associated with hyperdopaminergia in stage 1, *hypodopaminergic* function decreases behavioral plasticity, driving inflexibility and compulsivity. This gives rise to stage 2, when established AN behaviors are “locked in” and rendered resistant to change (2).

In this hypodopaminergic state, dopamine receptors likely upregulate expression and sensitivity (110–114), creating a physiological state of low basal dopamine concomitant with sensitized responses to phasic dopamine activity. Reduced basal/tonic dopamine coupled with enhanced receptor sensitivity has been described with chronic food restriction by Carr and colleagues [reviewed in Carr (50)] and proposed by Frank et al. (115) to play an important role in AN. Sensitization of D2R in particular has received attention in AN. Interestingly, the stimulation of D2R differentially affects cognitive flexibility based on levels of basal dopamine (116, 117), such that D2R activation improves cognitive flexibility when dopamine is low but impairs flexibility when dopamine is high. Consequently, differential D2R effects arising from different basal dopamine conditions may contribute importantly to changes in behavioral flexibility as individuals progress from stage 1 to stage 2 of AN.

The hypothesized dopamine reversal is likely driven by chronic HPA activation. Acute stress increases but chronic stress decreases dopamine function (42), possibly causing dopamine cell loss (70). As the HPA axis is a master orchestrator, these contrasting effects of acute and chronic stress can mediate reversals in other systems as well. For example, BDNF is increased with acute and decreased with chronic stress (118). Thus, chronic HPA activation resulting from persistent caloric restriction and low body weight may reverse neuroadaptations driving stage 1 and initiate a cascade of long-term adaptations, generating an entirely different profile of changes in stage 2.

## Mechanisms

Data supporting our proposed escalating spiral in dopamine in stage 1 is strong. Caloric restriction, exercise, stress, enhanced insulin sensitivity, decreased leptin, increased ghrelin and increased orexin can all enhance dopamine function, as outlined in Table 1. Furthermore, there is evidence demonstrating synergy between these modulators; for example, both exercise and caloric restriction enhance insulin sensitivity. Progressive adaptations over time are more difficult to characterize, as is observed in the literature on obesity (108, 119), addiction (41) and stress (42, 68). In each field, there are differences between acute and chronic conditions, often suggesting a reversal from enhanced to diminished dopamine function, as proposed here for AN. Studying progressive changes requires looking across longer periods of time, which can be challenging in research studies, including determining what amount of time constitutes ‘chronic.' This issue of time course is compounded by the fact that many adaptations not only interact, but potentially undergo long-term changes at different rates and induce compensatory adaptations, which may themselves arise at different times. In our hypothesis, we suggest that in stage 2, chronic caloric deficits induce a *cascade* of neuroadaptations (Figure 1B), but do not speculate on the detailed order of these adaptations, their interactions or compensatory changes, as this is beyond the scope of the current perspective. Instead, in Table 1 we include data supporting the notion that there is a reversal of adaptations in chronic conditions that may underlie stage 2. With the exception of altered D2 binding observed by Frank and colleagues (13), specific mechanisms affecting dopamine (i.e., synthesis, reuptake, storage, synaptic plasticity of inputs, burst activity) have not been characterized in AN patients.

## Implications

If each stage is mediated by different underlying neuroadaptations, then pharmacological treatments might differ by stage. In stage 1, drugs that prevent dopamine escalation (e.g., tetrabenazine) may slow development of the disorder, facilitating preventative cognitive-behavioral interventions. In stage 2, drugs that *enhance* dopamine might promote the behavioral flexibility needed to change entrenched behaviors. Conversely, treatments that modulate dopamine in the wrong direction would be predicted to be ineffective and could even be detrimental and facilitate the disorder. Patients are most likely to receive an AN diagnosis in our stage 2 when dopamine is low, potentially explaining the lack of efficacy of dopamine antagonists in AN treatment (30, 120, 121). In contrast, Frank and colleagues proposed using dopamine agonists to treat AN (115), arguing receptor activation would downregulate receptor hypersensitivity arising from diminished dopamine. As a partial D2R agonist, aripiprazole would remediate low basal DA through its agonist properties while the reduced (partial) activation would mitigate super sensitized responses to phasic DA, putatively normalizing the dynamic range of dopamine signaling. Consistent with these ideas, aripiprazole has been shown to promote weight gain in AN (122, 123). Interestingly, aripiprazole may have utility in stage 1 as well where its partial agonist properties may counteract escalating increases in dopamine. Notably, the finding that D2R acting drugs impair cognitive flexibility when dopamine is high (116, 117) calls into question whether decreasing plasticity during stage 1 would be advantageous (slowing development of AN behaviors) or detrimental (reducing impact of CBT intervention), possibilities that need to be investigated.

Given that few prospective studies have been conducted, most of what is known about AN is based on studying individuals in our putative stage 2, while stage 1 remains relatively uncharted territory. If our hypothesis is correct, stage 1 reflects a period of high behavioral plasticity providing a window of opportunity where interventions may be more successful, even preventative. The challenge is identifying those in stage 1 where evidence of AN may not yet fulfill diagnostic requirements; that is, differentiating individuals who are simply successful dieters from those who will develop AN. Prospective studies of dieters that identify factors predictive of AN could lead to diagnostic tools, ideally biomarkers, for early detection of AN in our proposed stage 1 [e.g., (122)]. These predictive factors may also apply to those who develop AN following unintentional weight loss. Furthermore, such prospective studies in dieters could provide insight into factors predicting (non-AN) success vs. failure in establishing sustained weight loss behaviors.

Our hypothesis would suggest any gene variants that regulate how the dopamine system responds to weight loss, exercise or chronic stress may in turn modify AN risk. This might include dopamine-related genes or genes of other systems– such as leptin, ghrelin, HPA axis, insulin– that modulate dopamine. Risk modification may be stage specific such that some variants may render an individual more likely to develop AN (stage 1) or more likely to progress to severe, persisting AN (stage 2).

## Testing the Hypothesis

Our hypothesis can be tested in humans by measuring dopamine (e.g., PET) prior to and at timepoints following initiation of dieting in a prospective study. We predict that weight loss resulting from dieting, particularly in combination with exercise, will increase dopamine function in most participants, but those who develop AN will show a more pronounced dopamine increase. We predict that those with AN who develop behavioral rigidity and treatment resistance will subsequently exhibit impaired dopamine, while those who are successfully treated will not. Moreover, we predict that behavioral change in successful, non-AN dieters might be associated with a more modest, time-limited increase in dopamine, while unsuccessful dieters will exhibit a minimal change in dopamine during diet adherence. Impaired dopamine found in obese individuals (108) may render them less capable of upregulating dopamine and establishing new weight loss behaviors. Though conceptually straightforward, such human studies can be challenging. Alternatively, in preclinical studies dopamine can be measured in awake-behaving rodents in the activity-based anorexia model (38). If our hypothesis is correct, we expect vulnerable mice to show dopamine escalation followed by impairment, while resilient mice may show a modest, time-limited increase in dopamine, as proposed in successful dieters.

## Conclusions

Our hypothesis is specific to AN but reflects a broader pattern common to disorders marked by compulsive behavior^1^, including addiction, obesity, and possibly other eating disorders (130). When an individual repeatedly engages in behavior that releases dopamine, this dopamine activation enhances behavioral plasticity, that in turn facilitates the reorganization of behavior around those dopamine releasing activities. Over time, these behaviors induce neuroadaptations that impair dopamine, reducing behavioral plasticity and entrenching the reorganized behaviors. If correct, our hypothesis would have broad implications for understanding and treating many behavioral disorders that incur profound social and economic costs.

## Data Availability Statement

The original contributions presented in the study are included in the article/supplementary material, further inquiries can be directed to the corresponding author/s.

## Author Contributions

All authors listed have made a substantial, direct, and intellectual contribution to the work and approved it for publication.

## Funding

This work was supported by NIDA, DA046058 (JB), NIMH, R21MH114182 (NB), US National Institute on Minority Health and Health Disparities of the NIH, G12MD007599 (NB), PSC-CUNY Awards jointly funded by the Professional Staff Congress and The City University of New York (JB and NB) and a Klarman Family Foundation Eating Disorders Grant (JB and NB).

## Conflict of Interest

The authors declare that the research was conducted in the absence of any commercial or financial relationships that could be construed as a potential conflict of interest.

## Publisher's Note

All claims expressed in this article are solely those of the authors and do not necessarily represent those of their affiliated organizations, or those of the publisher, the editors and the reviewers. Any product that may be evaluated in this article, or claim that may be made by its manufacturer, is not guaranteed or endorsed by the publisher.

## Footnotes

^1^Here we refer to disorders that arise from behaviors that induce dopamine release, such as drug taking, overeating, and gambling. We do not include obsessive compulsive disorder (OCD). Compulsive behaviors in OCD may be reinforced by providing relief from anxiety surrounding intrusive, obsessive thoughts. However, the root of the disorder likely lies in failure of executive inhibitory control to stop the obsessive thoughts that generate the compulsive behavior rather than in the reinforcement of the behavior *per se* (124). While there is evidence suggesting linkage between OCD and AN (125), pharmacological treatments for OCD have not been effective in AN (126, 127) and some have argued against this OCD-AN linkage (128, 129).
